# Common Glomerular Diseases in Adult Jordanians: A Single-Center Experience

**DOI:** 10.1155/2022/5292635

**Published:** 2022-07-04

**Authors:** Ahmed Sheyyab, Mohammad Al-thnaibat, Aseel A. Zghayer, Jafar Alsheyyab, Radi Hamed

**Affiliations:** ^1^Department of Internal Medicine, Faculty of Medicine, The Hashemite University, Zarqa 13133, Jordan; ^2^Department of Pediatrics, Faculty of Medicine, The Hashemite University, Zarqa 13133, Jordan

## Abstract

The pattern of glomerular diseases has been reported previously with contradictory results. Our primary objective is to assess the relative frequencies of glomerular disease in adult Jordanians and compare it with other institutes. A secondary objective is to assess the contribution of environmental factors, in an industrial city Zarqa, to kidney disease patterns. *Methods*. A retrospective study was conducted at a referral hospital center in the central region of Jordan. Assessment of native kidney biopsies, pathological reports, and the patients' characteristics were obtained from electronic medical records. *Results*. Our study assessed a total of 178 biopsies, of which 106 patients were included achieving the age criterion for adults. The mean age of our patient was 34 ± 12.7. The number of females (53.7%) was slightly more than males (46.3%). The average creatinine at presentation was 198 umol/L. Almost half of the patients had mild renal impairment (50.9%), while the remaining were divided between moderate (26.1%) and severe (27.3%). The indications of kidney biopsy were proteinuria (11.3%), proteinuria (54.7%), and unexplained renal impairment (34%). The leading common glomerular diseases were represented as a group with a relative frequency ranging between 11% and 13%. Both IgA nephropathy (13.2%) and lupus nephritis (12.2%) were the top conditions causing the nephritic syndrome, while focal segmental glomerulosclerosis (12.2%) and minimal change disease (11.3%) were the conditions leading to nephrotic syndrome. Our secondary analysis showed nonstatistically significantly higher glomerular filtration rates in the city of Zarqa, when compared to Amman (median 94 and 54, respectively, *U* = 469.5, *r* = 0.08, *p* = 0.491). Additionally, Zarqa had higher frequency rates of interstitial/tubular nephritis (*χ*^2^(1) = 1.17, *p* = 0.279, Cramér's *V* = 0.13. *Conclusion*. Common glomerular diseases, as reported internationally, were common among Jordanian adults.

## 1. Introduction

Glomerular diseases represent a diverse group of inflammatory disorders, which can be reversible when treated promptly. National epidemiological data is useful to guide clinicians to prevalent glomerular diseases and expected pathological patterns.

Multiple studies have been performed in Jordan with conflicting results. Two studies reported membranoproliferative glomerulonephritis (MPGN) as the most common glomerulonephritis [1, 2]. While another recent study reported the most common cause of GN as membranous GN [3]. In addition, these former studies reported some GN types, (i.e., IgA nephropathy), at a relatively lower rate than the expected rate at 3.4% and 8.1% [1, 3]. This contradicts with international data, which consistently reports IgA as the most common GN worldwide [4]. This variation in reporting of GN patterns raises questions regarding the generalizability of these patterns on the Jordanian population as a whole. This requires more research from different institutes, which could confirm their results.

Our study aims to assess patterns of glomerular diseases at our institute, compare them with other institutes, and provide possible explanations for glomerulonephritis pattern variations in Jordan and internationally.

## 2. Study Design and Methodology

Our study is a cross-sectional retrospective study conducted at Prince Hamzah Hospital, a tertiary hospital located in the central region of Jordan, Amman. All patients who had kidney biopsy reports from the period between January 1, 2013, till December 31, 2020, were assessed for inclusion in the study. Our inclusion criteria included native kidney biopsies for adult patients (defined as ≥ 16 years). Our study excluded biopsies from kidney transplant patients and native biopsies for patients in the pediatric age group (<16 years old). A detailed chart review of the included patients was performed using an electronic medical record system (Hakeem Electric Health Solutions). The electronic chart review entailed patients' clinical notes, pathological reports, and laboratory data. The study was approved by the hospital's institutional review board (IRB), waiving the documentation of informed consent.

Analysis of data was performed using IBM SPSS statistics for Windows version 23.0. Statistical analysis was performed using both parametric testing for the analysis of entire study patients, *t*-test for independent samples, and the chi-square test, while the nonparametric test (i.e., Mann–Whitney *U* Test) was used for subgroup analysis. Missing data for continuous variables were imputed depending on the average observations. For categorical variables, missing data were equally reported as “missing” when the rates of missing data were >10% of their total observations.

The glomerular filtration rate (GFR) was estimated using the chronic kidney disease epidemiology collaboration (CKD-EPI) equation. The acuity of kidney disease was assessed depending on baseline serum creatinine and adopted from the RIFLE (risk, injury, failure, loss of kidney function, and end-stage kidney disease) classification [5]. Acute injury was defined as a 25% increase in serum creatinine which occurred over days to weeks, while increase in creatinine levels slowly over months or years was classified as chronic kidney disease.

## 3. Results

Our study assessed a total of 178 native kidney biopsies, 106 adult patients were included, and 72 in the pediatric age group were separated in another report. The majority of our patients (91 patients) were young adults of age ranging from 20–49 years. There was a slight predominance of female patients (53.7%) over males (46.3%). Males were comparable to female patients in most aspects except that males had higher serum creatinine (umol/L) at presentation (274 and 123, respectively, *p*=0.001). A detailed comparison of the characteristics between male patients and female patients is presented in Table 1.

In the overall studied patients, the creatinine levels at the time of presentation average at 198 umol/L, which corresponds to an estimated glomerular filtration rate of 66.5 ml/min/1.73 m^2^. This calculation excluded two outlier data observations which were above 900 umol/L. The impairment of kidney function was classified as acute in 30.1% of patients, or chronic in 69.8%. The severity of renal impairment at presentation was considered mild (GFR 60–120 ml/min/1.73 m^2^) in 50.9%, moderate (estimated GFR 30–60 ml/min/1.73 m^2^) in 21.6%, and severe (estimated GFR <30 ml/min/1.73 m^2^) in 27.3%. The indications of kidney biopsies were proteinuria in 58%, hematuria with proteinuria in 11.3%, and unexplained renal impairment in 34%. The degree of proteinuria was present in variable proportions, 49% of patients in the subnephrotic range, 38.6% the in nephrotic range, and no evidence of proteinuria was found in 12%.

Overall assessment of kidney biopsies revealed that primary GN was reported in 81.1% of our studied cohort. In contrast, secondary GN was reported in 13.2% of patients. The disease biopsies were classified as nephritic syndrome (40%), nephrotic syndrome (32%), and nonglomerular (28.3%). Assessment of the types of GN demonstrated close frequency rates across different types of glomerulonephritis, with mainly four glomerular diseases achieving the highest rates ranging between 11 and 13%. IgA nephropathy (13.2%) and lupus nephritis (12.2%) were the highest two conditions causing the nephritic syndrome. Whereas, focal segmental sclerosis (11.3%) and minimal change disease (12.2%) were the top two entities leading to nephrotic syndrome.

**Figure 1:**
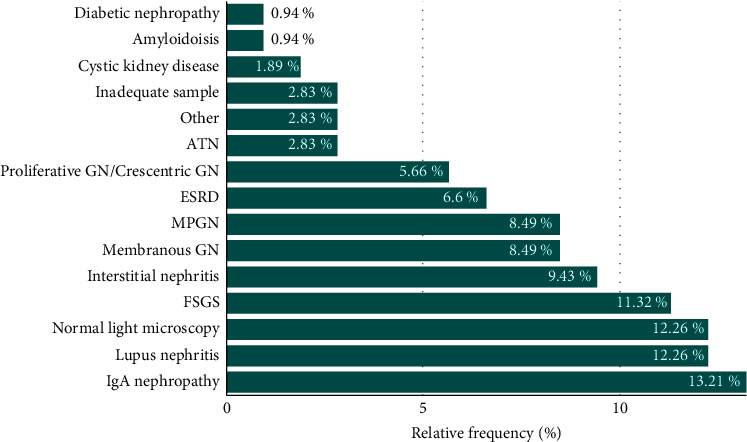
Relative frequencies of glomerular diseases at Prince Hamzah Hospital.

Glomerular conditions reported among the intermediate group which were ranked as the 5^th^ most common GN include membranous nephritis and membranoproliferative glomerulonephritis pattern (MPGN), which were reported equally in 9 patients (8.5%). The remaining biopsies included rare conditions, which are detailed in Figure 1, which demonstrates the relative frequencies of all kidney biopsies studied.

Irreversible kidney damage manifested in variable degrees. Diffuse glomerulosclerosis with a final diagnosis of “End-Stage Renal Disease” was identified in 6.6% of patients. The remainder of patients had milder chronic changes, with global sclerosis present in 50% of patients, but segmental sclerosis was reported in only 11.3%. Interstitial fibrosis was reported in 24.5% of patients, while absence of fibrosis was reported only in 7.5% of patients. The remaining either had interstitial inflammation in 28.3% of patients, and 39.6% had missing description on the degree of fibrosis.

Tissue adequacy was assessed depending on multiple parameters. The average number of tissue cores obtained was approximately 2.1. Most samples of kidney biopsies were considered “technically adequate” except for 3 patients, in which the pathologist recommended repeating the performance of kidney biopsy. However, true tissue adequacy criteria were not achieved for focal segmental glomerulosclerosis (>20 glomeruli), with an average glomerulus of 11.1 glomeruli.

**Figure 2:**
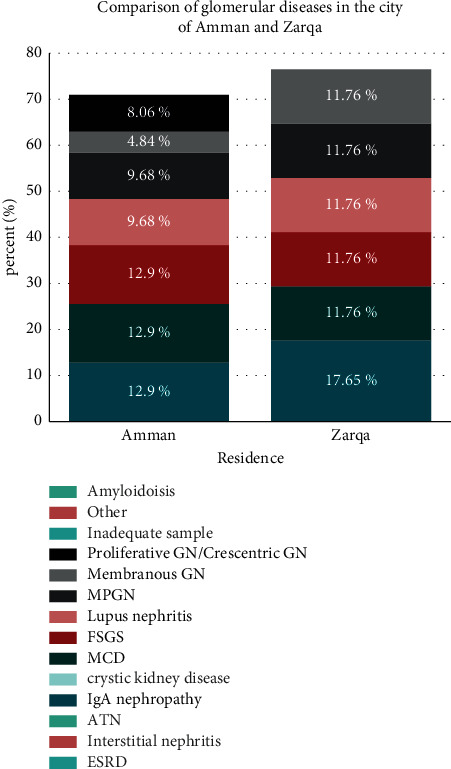
Relative frequencies of glomerular disease in a subgroup of patients residing in two different cities, Amman and Zarqa.

A subgroup analysis of patients residing in the central region of Jordan showed that patients in the city of Amman (62 patients) had lower GFR at presentation than patients from Zarqa city (17 patients), a finding that was not statistically significant (median 54 and 94, respectively, *U* = 469.5, *r* = 0.08, *p*=0.491). The rates of glomerular diseases and interstitial disease were different in Amman residents (87.2% and 12.7%, respectively) compared to Zarqa residents (76.5% and 23.4%, respectively). These difference was not statistically significant (*χ*^2^(1) = 1.17, *p*=0.279, Cramér's *V* = 0.13). The types of glomerular diseases in the two cities were reported with a high degree of resemblance, as demonstrated in Figure 2.

## 4. Discussion

Our results demonstrated that common glomerular diseases (such as IgA nephropathy) are common among Jordanian patients; this is evident by demonstrating IgA nephropathy as the most common disease overall with an incidence of 13.2%. This is consistent with international data which indicate IgA nephropathy as the most prevalent GN worldwide [4]. However, Jordanian prevalence data of IgA nephropathy was previously reported at noticeably lower rates than the expected rate of 3.4% and 8.1% [1, 3]. These differences can be explained by under-detection of the disease. First, the differences in local biopsy practices, particularly when the biopsy is averted for asymptomatic patients or subclinical diseases. Second, patients with severe forms of IgA nephropathy may be missed due to rapid progression of the disease to ESRD.

In patients with nephrotic syndrome, FSGS and normal biopsies (i.e., clinically diagnosed as minimal change disease), both acheived the highest frequency rates. These findings are consistent with previous local reports, which demonstrated FSGS as the second most frequent GN overall [1–3, 6]. It is possible that estimates of FSGS incidence are actually higher than those that resulted in our study, given the focal nature of the disease and our failure to achieve the tissue adequacy criteria (>2 cores and >20 glomeruli). More importantly, our study demonstrates the difficulty to obtain the adequate number of glomeruli from two tissue core samples. Our average, the number of glomeruli of 11, mostly obtained from 2 cores, contained much lower glomeruli than the number needed to detect FSGS. This data strongly suggests the need to obtain at least 3 samples that might be necessary to achieve tissue adequacy. This practice could help in avoiding repeating the procedure and lead to earlier detection of this condition.

The high incidence of FSGS in Jordan is an important finding which requires an assessment of factors contributing to this disease. We suspect that the high incidence of FSGS among Jordanians is related to genetic factors. In a Jordanian pediatric cohort of FSGS, high rates of familial FSGS reaching as high as 20.2% were revealed [7]. We believe that genetic predisposition is likely to be an influential factor in adult patients. This requires genetic studies in families with FSGS to confirm this assumption and identify underlying genetic mutations.

Our study provided important clues to what are the common glomerular diseases among the Jordanian population. Also, combining the results of studies can aid in improving our estimations of prevalence. However, careful interpretation of minor differences of disease frequency between studies is key to avoiding misinterpretation. For example, we reported membranous GN among the middle group of GN at 8.5%. Our result was consistent with some of the Jordanian studies, which reported membranous GN at 7.9% and 6.3% [1, 2], but contradicted another report by Farah et al, which showed membranous GN as the most common GN [3]. The former author postulated that a possible shift of the GN pattern, from MGN to MPGN, may have occurred with time. Another explanation is that a shift in biopsy results might not necessarily reflect a true incidence shift, but rather reflect differences in the detection rates of MPGN. Important factors, such as acquired center experiences with time, play a role, which includes the experiences gained by nephrologists to perform biopsies at a lower threshold and the experience gained by pathologists in the assessment of glomerular diseases [8]. This explanation is supported by two studies, which reported wide variability of pathological reporting of kidney biopsies [9, 10]. The second explanation is, it is given that increased detection rates of other common glomerular diseases may affect the proportion of the less common disease. In a study performed at the University of North Carolina, a marked increased frequency of diabetic nephropathy from 5.5% to 14.2% was revealed, which was accompanied with a decrease in the frequency of MPGN from 4.5 to 3.3 [11].

Our study compared the largest two cities in Jordan. Amman, the capital of Jordan, is a modern and the largest populated city in Jordan with an estimated 4.5 million [12]. Zarqa is an industrial city with a population of 1.5 million with lower socioeconomic living conditions [13]. Comparison between those cities revealed the contribution of environmental factors and socioeconomic influences on kidney disease. Environmental exposure, industrial exposure to heavy metals (e.g., arsenic and cadmium), and exposure to hydrocarbons have been linked to glomerular diseases [14, 15]. Our results showed increased rates of interstitial/tubular disease in Zarqa when compared to Amman, a finding that hints the possible contribution of environmental factors (i.e., heavy metal exposure). This finding needs confirmation in larger studies.

Our study had limitations. First, our study had a small sample size which reduced its power to detect statistical differences. Second, electron microscopy reporting was poorly documented, which limited our conclusions regarding reports of normal light microscopy. Third, our study had a low proportion of elderly patients and diabetic patients biopsied, which could affect the relative frequencies of other glomerular diseases.

In summary, our study supports the notion that common glomerular diseases reported internationally, IgA and FSGS, are also common in Jordan. Our findings hint at possible variability in kidney disease severity and patterns between different cities, although this requires confirmation in future studies. [11]

## Figures and Tables

**Table 1:** Demographic and clinical characteristics of the studied patients with comparison in their features between male and female patients.

	Overall (*n* = 106)	Male (*n* = 49)	Female (*n* = 57)	
Age (years)	34 ± 12.7	35.02 ± 11.3	33.14 ± 13.8	*p*=0.45
Creatinine (umol/L)	198 ± 232	279.94 ± 288	129.02 ± 139.7	*p*=0.001
GFR at presentation (ml/Min/1.73 m2)	66 ± 43	62.37 ± 45	70.14 ± 41.9	*p*=0.36
Renal disease severity				
Mild	54 (50.9%)	21 (38.8%)	33 (61.2%)	
Moderate	23 (21.6%)	15 (51.7%)	14 (48.3%)	
Severe	29 (27.3%)	13 (56.5%)	10 (43.4%)	*p*=0.286
Acuity of renal impairment				
Acute	32 (30.1%)	15 (47%)	17 (53%)	
Chronic	74 (69.8%)	34 (46%)	40 (54%)	*p*=0.93
Indication of kidney biopsy				
Hematuria with proteinuria	12 (11.3%)	7 (58.3%)	5 (41.7%)	
Unexplained renal impairment	32 (33.9%)	11 (30.5%)	25 (69.5%)	
Proteinuria alone	58 (54.7%)	31 (53.4%)	27 (46.5%)	*p*=0.065
Hematuria				
Presence of hematuria	49 (53.7%)	26 (53%)	23 (47%)	
No evidence of hematuria	57 (46.2%)	23 (40.3%)	34 (59.7%)	*p*=0.191
Degree of proteinuria				
Nephrotic range	41 (38.6%)	23 (56%)	18 (44%)	
Subnephrotic range	52 (49%)	22 (42.3%)	30 (57.6%)	
No evidence of proteinuria	13 (12.2%)	4 (30.7%)	9 (69.2)	*p*=0.204

## Data Availability

Study data are available and would be provided upon request. Requests for data access can be submitted and sent by e-mail, at the following address: ahmedm_ya@hu.edu.jo.
